# Enhanced Thermal Stability of Mesoporous Carbon Microbeads-Based Lithium-Ion Batteries by Propargyl Methacrylate as Electrolyte Additive

**DOI:** 10.3390/polym14214491

**Published:** 2022-10-24

**Authors:** Yu-Ruei Kung, Jing-Tang Su, Chiung-Cheng Huang, Yaoming Xiao, Jeng-Yu Lin

**Affiliations:** 1 Department of Chemical Engineering and Biotechnology, Tatung University, Taipei 104327, Taiwan; 2 College of Chemical Engineering and Materials Science, Quanzhou Normal University, Quanzhou 362046, China; 3 Department of Chemical and Materials Engineering, Tunghai University, Taichung City 407224, Taiwan

**Keywords:** propargyl methacrylate (PMA), mesoporous carbon microbeads (MCMB), solid electrolyte interface (SEI)

## Abstract

In this current work, propargyl methacrylate (PMA) was successfully adopted to be an efficient electrolyte additive to stabilize the formation of a solid electrolyte interface (SEI) layer on mesoporous carbon microbeads (MCMB) in Li-ion batteries, especially at elevated temperatures. According to a series of material and electrochemical characterizations, the optimized concentration of PMA additive in the electrolyte was found to be 0.5 wt.%. The MCMB electrode cycled with the optimized 0.5 wt.% PMA-containing electrolyte exhibited impressive capacity retention of 90.3% after 50 cycles at 0.1C at elevated temperature, which was remarkably higher than that using the PMA-free electrolyte (83.5%). The improved electrochemical stability at elevated temperature could be ascribed to the rapid formation of stable and thin SEI layer on MCMB surface, which were investigated and suggested to be formed via PMA copolymerization reactions.

## 1. Introduction

Lithium-ion battery (LIB) has been turned into one of the promising energy storage devices due to its excellent performance related to energy density, lifetime, ease of use, and low cost of maintenance over other traditional batteries [1,2,3]. Nevertheless, conventional graphite-based anodes still suffer from severe issues in the significant concerns of safety, cyclic performance, and high-rate performance although they commonly possess high energy density, low operating potential, and long cycle life during the charge and discharge process. The low working potential of graphite generally results in the formation of dendrites due to the continuous lithium deposition, which would therefore cause the safety issue during charging and discharge. During the initial charging/discharging process, an electrically insulating and ionically conductive surface passive film is formed by electrolyte decomposition, which is named a solid electrolyte interface (SEI) [4]. SEI layer formation process would suppress the efficiency of LIBs since it consumes a considerable part of the anode material and electrolyte. However, it also effectively prevents the electrolyte from further reduction reaction on the surface of the anode, thereby protecting the structure of the graphite anode and improving the cycling life and safety of the graphite-based LIBs [5]. Since the properties of SEI film would be necessary for achieving better battery performance, it is necessary to form a suitable SEI passive layer. Consequently, great deals of electrolyte additives have been used in the electrolyte to improve the quality of the SEI passive film and efficiently suppress the electrolyte decomposition [6,7].

Several electrolyte additive systems have been widely reported for LIB application for improving their cycliability and/or coulombic efficiency as collected and illustrated in Table S1, such as vinylene carbonate (VC) [8,9,10,11,12], borate/boronic acid [13,14], vinyl acetate (VA) [15], propane sultone (PS) [16], ethylene sulfite (ES) [17,18], propargyl methanesulfonate (PMS) [18,19,20] and other sulfur-containing compounds [21,22,23,24,25]. Most of the electrolyte additive compounds are with sulfonyl (-SO_2_-), carbonyl (-C=O), and vinyl (CH_2_=CH_2_) functional groups for tuning the polarity. In general, electron-withdrawing oxygen and sulfur units make the vinyl group as electrophilic, which facilitates the reduction process to stabilize the formation of the SEI film [26,27]. Among the various electrolyte additive materials, VC is found to produce a durable, stable, cohesive network SEI formation, which can efficiently prevent from cracking and continuously re-exposing of the active materials to the electrolyte during charging and discharging processes [28]. As forming the SEI layer on graphite electrode by introducing PMS electrolyte additive agent, the layer consists of organic components produced by the decomposition of the alkyne moiety, indicating that the related low contents of inorganic components, such as LiF, Li_2_CO_3_ inside the SEI layer [15]. Owing to the dense structure and low impedance characterization of the SEI layer, the freshly formed SEI by alkyne additive could provide efficient electrochemical behavior as a passivated layer in Li-ion batteries. They also observed that the SEI layer grown in the presence of VC can efficiently facilitate suppressing the salt anion (PF_6_) decomposition. Normally unsaturated terminal functionality like vinyl, allyl, and propargyl groups can efficiently promote the polymerization of electrolyte additives on the electrode surface and thereby, increase the resistance. This process generally forms a passive film or a network structure on the electrode surface to improve the long-term performance of LIBs. The formation of SEI film can efficiently suppress the electrolyte-electrode reaction, which results in reduced gaseous ethylene generation during elevated temperature storage and cycling operations.

**Scheme 1:**
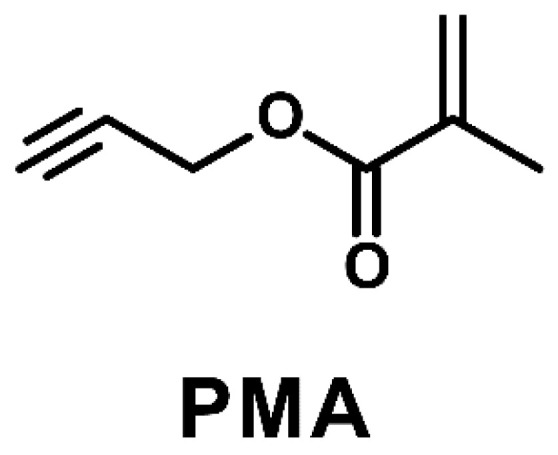
Chemical structure of propargyl methacrylate (PMA).

By taking the advantage of electrolyte additives, we systemically investigated the role of propargyl methacrylate (PMA) with a redox-active functional group as an efficient electrolyte additive (Scheme 1) to address the formation of SEI films on MCMB anodes and improve their long-term electrochemical stability, especially at elevated temperature. Moreover, the polymerization mechanism of the PMA additive during cycling operation was investigated in this research.

## 2. Materials and Methods

### 2.1. Materials

MCMB and PMA were purchased from China Steel Chemical Corporation, Taiwan. Conductive carbon black, Super P, polyvinylidene fluoride (PVDF) Kynar 7200 were purchased from Arkema Technical Polymers. Anhydrous *N*-methyl-2-pyrrollidine (NMP) was obtained from Merck. Ethylene carbonate (EC) and diethyl carbonate (DEC) were mixed with 1.0 M LiPF_6_ obtained from Formosa Plastics Group, Taiwan. All reagents, as mentioned above, were in the analytical grade and used directly without any further purification.

### 2.2. Fabrication of Coin Cells

The MCMB electrodes were fabricated by stirring continuously with 85 wt.% MCMB, 10 wt.% super P and 5 wt.% PVDF in NMP for 2 h, and subsequently the well-mixed slurry was evenly coated on a thin Cu foil by using a blade-coating method with an automatic coater, and then dried at 120 °C for 12 h in a vacuum to remove the most residual NMP solvent and water. Prior to the assembly of the coin cells, the coated MCMB foils were punched into circular electrodes of 10 mm in diameter. Afterward, the 2032-type coin cells were assembled using the MCMB electrodes as the anodes and the lithium metal foils as cathodes in addition to Celgard 2500 monolayer polypropylene (PP) was used as the membrane separator. The assembly of the coin cells was performed in a glove box filled with argon. The as-fabricated coin cells were kept at room temperature for 12 h to ensure the complete impregnation of the electrodes and separators with electrolyte. The base electrolyte solution without PMA additive was composed of 1M LiPF_6_ in ethylene EC/DEC with a volume ratio of 1:1. Additionally, the base electrolytes containing different weight ratios of PMA from 0.5 to 2 wt.% were prepared and employed.

### 2.3. Methods

The morphology and microstructure of the samples were analysed by field-emission scanning electron microscope (FE-SEM, JEOL JSM-7000F, JEOL Ltd., Tokyo, Japan). The chemical bonding of the samples was examined via an attenuated total reflection–Fourier transform infrared (ATR-FTIR) spectroscopy (Jasco 6700, Jasco Inc., Tokyo, Japan). The surface chemical state was characterized using X-ray photoelectron spectroscopy (XPS, VG ESCA Scientific Theta Probe). PHI Quantera SXM X-ray photoelectron spectrometer (ULVAC-PHI Inc., Kanagawa, Japan) with an Al Ka X-ray radiation. The negative ion depth profiling method from the secondary ion mass spectrometry (SIMS) analyzed the pristine and cycled electrode materials. A pulsed 30 keV Bi^+^ primary ion source was used on 100 × 100 µm area for testing with the current density of 1 pA for all electrodes. Depth profiling was measured by sputtering with 1 keV Cs^+^ ion gun, which produces the 40 nA target current over a 260 × 260 µm area for all electrodes. The galvanostatic charge/discharge tests of the assembled coin cells were performed with a battery autotest system (BAT-750B, AcuTech Systems Co., Ltd.) between 0.005 V and 2 V at room temperature (25 °C) and elevated temperature (55 °C). The cyclic voltammetry (CV) was conducted using a CHI 614D electrochemical workstation at a sweep rate of 0.01 mV s^−1^ between 0.005 and 2 V. Electrochemical impedance spectroscopy (EIS) was performed using a Zahner IM6 potentiostat in the frequency range of 100 kHz to 10 MHz with an AC amplitude of 10 mV.

## 3. Results and Discussion

**Figure 1:**
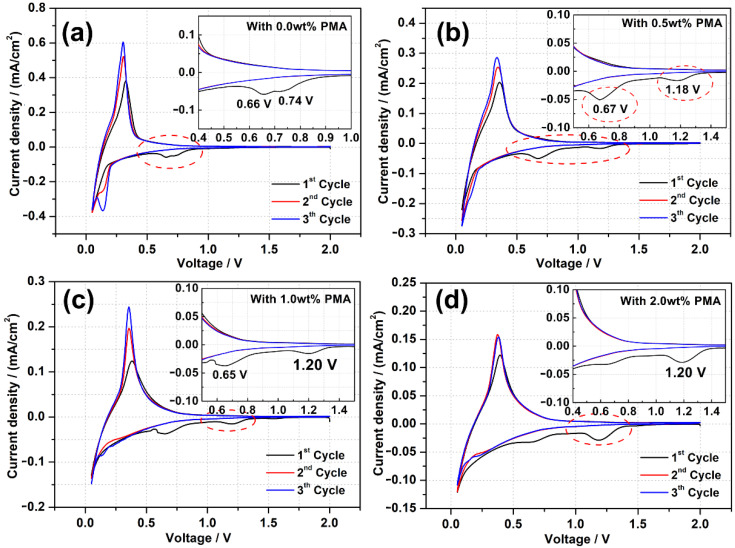
CV curves and their enlarged regions of the MCMB electrodes recorded in the base electrolyte containing (**a**) 0.0 wt.%, (**b**) 0.5 wt.%, (**c**) 1.0 wt.% and (**d**) 2.0 wt.% of PMA additive at a scan rate of 0.1 mV/s.

To investigate the SEI formation of the PMA-modified MCMB electrodes with different concentrations, CV experiments were conducted and depicted in Figure 1. The PMA-free system in Figure 1a shows an obviously irreversible cathodic peak generated around 0.2 V after the first cycle scan, which represents the reasonable processes of lithium intercalation into the MCMB electrode [29]. Figure 1b–d shows all the CV curves of MCMB electrodes measured in the base electrolyte containing different concentrations of PMA additive. As represented in Figure 1a, the well-defined peaks appeared at 0.66 and 0.73 V vs. Li^+^/Li in the first cycle and then disappeared in the subsequent cycles due to the rapid SEI formation from EC and DEC electrolytes [11]. More importantly, the cathodic peak below 0.2 V and anodic peak between 0.2 V to 0.5 V are representative peaks corresponding to the lithium intercalation into and de-intercalation from the MCMB electrode, respectively [30]. All PMA-modified MCMB electrodes represent the same peak caused by SEI formation at around 0.66 V in addition to the peak at around 1.20 V. The intensities of peaks at 1.20 V increase with the amount of PMA, confirming that it should be related to the electrochemical reduction behavior of PMA additives. The anodic peak between 0.6 and 0.7 V can be assigned to the two-electron reduction of EC converted into Li_2_CO_3_ and ethylene as following equation 1 and the peak at 0.73 V is associated with EC one electron reduced into alkyl lithium carbonate (ROCO_2_Li) as shown in Equations (2) and (3), respectively [31].

EC + 2 e^−^ + 2 Li^+^ → Li_2_CO_3_ + CH_2_=CH_2_ (1)

EC + e^−^ + Li^+^ → [Li^+^ EC^−^] (2)

2 [Li^+^ EC^−^] → LiOCO_2_CH_2_CH_2_CO_2_OLi + CH_2_=CH_2_ (3)

On the other hand, an additional reduction peak at the higher potential of around 1.20 V vs. Li^+^/Li is observed for the MCMB anodes in the PMA-containing electrolytes, which can be ascribed to the initiation of SEI formation on the MCMB electrode surface due to the decomposition of PMA. However, the reduction peaks are found to be vanishhed in the consequent CV cycles. Since the reduction potential of PMA (1.20 V vs. Li^+^/Li) is higher than that of the EC/DEC based electrolyte (0.7 V vs. Li^+^/Li), indicating that PMA is preferentially reduced on the MCMB surface before the reduction of the EC/DEC based electrolyte. Moreover, the current density of the reduction peak due to the PMA decomposition at the first cycle is found to be increased with increasing the PMA concentration in the electrolyte. This signifies that more decomposition of PMA on MCMB surface could occur with increasing PMA concentration. On the other hand, the current density of the reduction peak derived from the decomposition of the EC/DEC based electrolyte considerably diminishes upon the presence of the PMA additive, which can dynamically retard the further decomposition of the EC/DEC electrolyte. Furthermore, it is observed that the cathodic peak below 0.2 V and an anodic peak between 0.2 V to 0.5 V become decreased with increasing the amount of PMA additive. This reveals that the thick SEI derived from the decomposition of high PMA concentration would be a barrier for reducing lithium intercalation/de-intercalation in the MCMB electrode.

**Figure 2:**
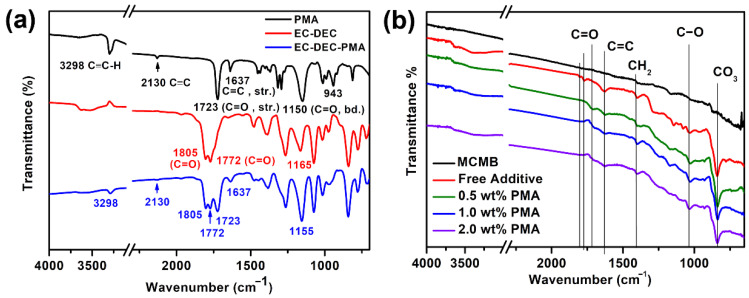
(**a**) ATR FT-IR spectra of PMA, EC/DEC electrolyte and EC/DEC containing PMA, and (**b**) ATR FT-IR spectra of fresh MCMB electrode and cycled MCMB electrode in the base electrolyte containing different PMA concentrations.

Figure 2a presents the ex-situ ATR FT-IR spectra of the pure PMA, EC/DEC electrolyte, and their mixture. As depicted in Figure 2a, the pure PMA reveals a strong peak at 3298 cm^−1^ and a medium-intensive peak near 2130 cm^−1^, which can be attributed to the alkyne H-C≡C and C≡C triple bond stretching vibrations, respectively. Moreover, the C=C stretching and =C-H out-of-plane bending vibration peaks at 1637 cm^−^^1^ and 943 cm^−1^, respectively, are associated with the alkene group of PMA. Furthermore, the vibration peak located at 1723 cm^−1^ is responsible for the acrylate ester functional group (O-C=O) of PMA. As for the EC/DEC electrolyte, the strong transmittance peaks at 1772 and 1805 cm^−1^ related to the C=O stretching vibrations from alkyl (for DEC) and cyclic (for EC) carbonate nature, can be obviously found, in addition to a vibration peak at around 1150 cm^−1^ correlated to the C-O bending vibrations. Figure 2b presents the ex-situ ATR FT-IR spectra of the fresh MCMB electrode and the cycled MCMB electrode in the electrolyte with various concentrations of PMA additive. As shown in Figure 2b, the three peaks located around 1700 to 1800 cm^−1^ for different carbonyl (C=O) groups, 1635 cm^−1^ (C=C), 1401 cm^−1^ (-CH_2_), 1034 cm^−1^ (C–O) are apparently found for all samples except the pristine MCMB electrode. These peaks mainly correspond to the formation of lithium alkyl carbonate (ROCO_2_Li) species [31]. Due to its relatively less content of PMA additive on the MCMB samples, the signals of alkyne group (H-C≡C and C≡C) are not easy to be observed in the FT-IR spectra, as shown in Figure 2b. Furthermore, the carbonyl groups (C=O, C-O) of EC and DEC are found to be a little shifted to the lower frequency for the cycled MCMB electrode with PMA additive. This phenomenon indicates that the introduction of PMA additive could enhance the solvation interactions of Li^+^ ions with the oxygen elements of carbonyl group (C=O, C-O) from EC/DEC-based electrolyte instead of the hydrogen bonding behaviors [32]. According to Aurbach et al. [11,33] Li_2_CO_3_ can be formed over the surface of Li electrode from decomposed open ring carbonates of electrolyte. They also demonstrated that EC and DEC could undergo two-electron redox reactions to form the nucleophilic carbonate intermediates and alkylene. Furthermore, unstable nucleophilic carbonates can readily react with the remained EC and DEC electrolytes to form alkyl carbonates, as described in Equations (4) and (5), respectively.

EC + DEC + 2e^−^ → CO_2_^−^+ CH_2_=CH_2_ (gas) (4)

CO_2_^−^ + DEC → CH_3_CH_2_(OCO_2_^−^)_2_ (5)

These dissociated carbonates combined with the Li^+^ ions would precipitate as well-known SEI thin films. Due to the low concentration of electrolyte near to counter electrode, the nucleophilic reaction can effectively be suppressed. Hence, the carbonate ion easily reacts with the Li^+^ ions to form a major surface species Li_2_CO_3_ and become as an important passive agent to stabilize the Li counter electrode. The ATR FT-IR spectra of the MCMB electrode cycled in the presence of PMA electrolyte additive show that the peaks associated with the ROCO_2_Li become much smaller than that in the absence of PMA additive, confirming that the formation of ROCO_2_Li corresponds to the two-electron electrochemical reduction process of EC, which exhibits a good agreement with the CV studies.

**Figure 3:**
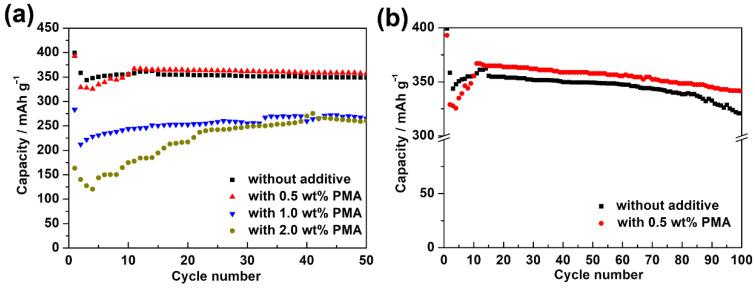
(**a**) Cycling performance of the MCMB electrodes charged and discharged in the base electrolyte containing various PMA concentrations at 0.1C for 50 cycles at room temperature. (**b**) The comparison of long-term cycling performance between the MCMB anodes tested in PMA-free and 0.5 wt.% PMA-containing electrolytes at 0.1 C for 100 cycles at room temperature.

Figure 3a presents the cycling performance of MCMB electrodes charged/discharged in the base electrolyte containing various PMA concentrations at 0.1 C for 50 cycles at room temperature. It is clearly seen that the MCMB anodes in PMA-free and 0.5 wt.% PMA-containing electrolytes displayed superior electrochemical stability compared to those tested in 1.0 wt.% and 2.0 wt.% PMA-containing electrolytes. To further investigate their long-term cycling performance, the galvanostatic charge-discharge tests were performed at 0.1 C for 100 cycles. As can be seen in Figure 3b, the discharge capacity of the MCMB electrode in the absence of PMA was decreased along with the significant capacity decay after 80 cycles. The capacity retention of the MCMB electrode in the PMA-free electrolyte was estimated as ca. 80.4%. However, with the introduction of 0.5 wt.% PMA in the base electrolyte, the capacity retention of the MCMB electrode was then improved to ca. 86.9%. This signifies that the introduction of 0.5 wt.% PMA can slightly improve the electrochemical stability of MCMB anodes when operated at room temperature.

**Figure 4:**
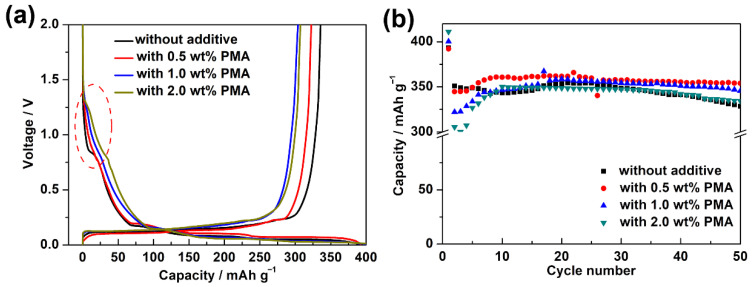
(**a**) Initial galvanostatic charge and discharge curves and (**b**) cycling performance of MCMB electrodes in the base electrolyte containing various PMA concentrations at 0.1C for 50 cycles at elevated temperature.

Figure 4a further shows the initial charge/discharge curves of the MCMB electrodes in the base electrolyte containing various PMA concentrations at elevated temperature. During the discharging process, the MCMB electrode in the additive-free electrolyte showed an obvious voltage plateau at around 0.75 V, which is highly associated with the reduction of the ECDEC-based electrolyte. However, that typical behavior almost disappeared in the charge/discharge curves of the cells in the presence of PMA additive. This confirms the findings from the CV curve that the PMA decomposition film could efficiently suppress the formation of SEI film due to the reduction of EC/DEC base electrolyte. The MCMB electrode with the PMA-free base electrolyte and 1.0 wt.% PMA-containing electrolyte exhibited a discharge capacity of 393.35 and 392.16 mAh g^−1^, respectively. When the PMA concentration was increased to 1.0 and 2.0 wt.%, the discharge capacity was further increased to 400.44 and 411.12 mAh g^−1^, respectively. The increased initial discharge capacity of the MCMB in the presence of high PMA concentration can be ascribed to the decomposition of PMA in the first discharge process. As depicted in Figure 4b, the cycling stability of the MCMB electrodes in the base electrolyte containing various PMA concentrations at elevated temperature was verified. It is worth noting that the capacity retention of the MCMB electrode was achieved up to 90.3% in the based electrolyte containing 0.5 wt.% PMA, which was significantly superior to that of using the additive-free electrolyte (83.5%). Nevertheless, the capacity retention of the MCMB electrode became decreased with further increasing the PMA concentration to 1.0 wt.% (86.3%) and 2.0 wt.% (81.3%). After 50 cycles, the discharge capacity of the MCMB electrode was found around 354.04 mAh g^−1^ in the base electrolyte containing 0.5 wt.% PMA, which was even significantly higher than that in the additive-free electrolyte (328.48 mAh g^−1^). As a result, the cycling performance of the MCMB electrode at elevated temperature could be remarkably improved when the base electrolyte contained 0.5 wt.% PMA.

**Figure 5:**
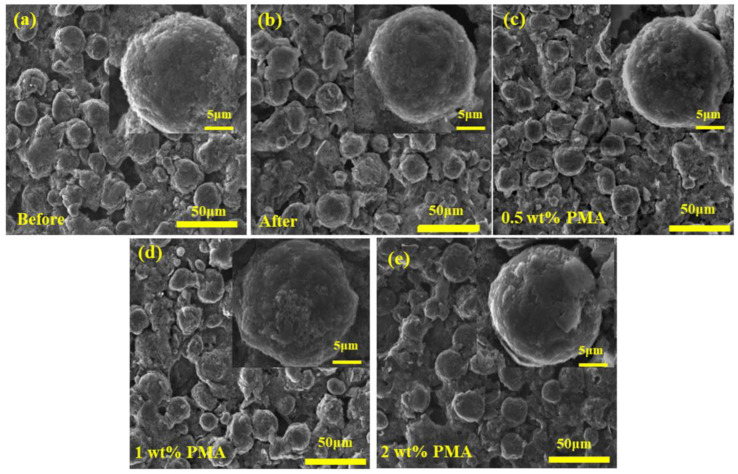
FE-SEM image of MCMB (**a**) before or after 3 cycles with (**b**) free additive, (**c**) 0.5 wt.%, (**d**) 1.0 wt.% and (**e**) 2.0 wt.% of PMA electrolyte additives.

To investigate the morphology, change of the MCMB electrode in the presence of PMA electrolyte additive, the FE-SEM images were taken for the MCMB electrodes charged/discharged for 3 cycles at a current density of 0.1 C at elevated temperature. As can be seen in Figure 5a,b, the surface morphology of the MCMB electrodes before and after charged/discharged in the PMA-free electrolyte did not have an obvious difference, suggesting that the growth of the SEI film from the decomposition of EC/DEC electrolyte is relatively slow. Nevertheless, the surface evolution of the MCMB electrodes was obviously observed when the PMA was introduced. It was found that the surface of the MCMB electrodes became smoother with increasing the PMA concentration, as depicted in Figure 5b–e. This confirms the decomposition of PMA additive, as shown in the CV studies. Figure S1 further presents the FE-SEM images of the MCMB electrodes after 100-cycle charged/discharged tests in the PMA-free and 0.5 wt.% PMA-containing electrolytes. As depicted in Figure S1a, the MCMB electrode in the absence of PMA additive reveals uneven and rough surface morphology, which could be due to the continued/uncontrolled growth during the prolonged cycles. In contrast, the smooth and compact SEI layer is found to be formed on the MCMB surface (Figure S1b) when operated in the 0.5 wt.% PMA-containing electrolyte. This suggests that the small amount of PMA additive could efficiently stabilize the SEI layer growth.

**Figure 6:**
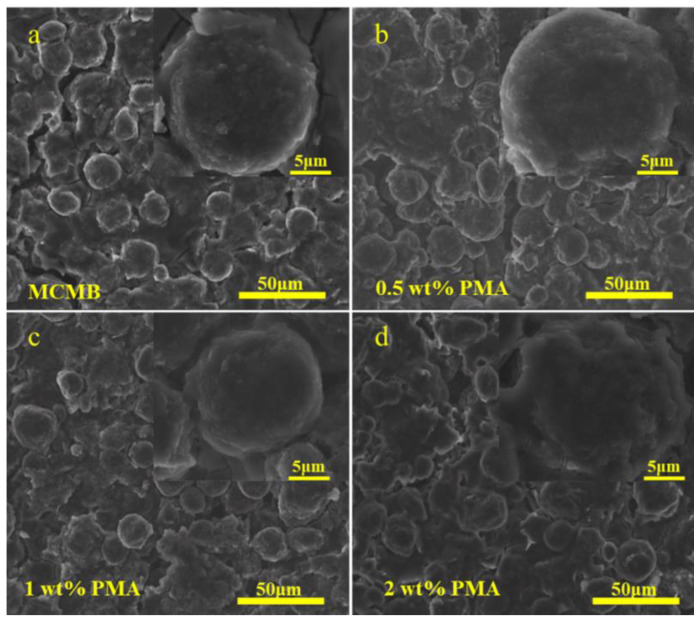
FE-SEM images of MCMB electrodes tested in the base electrolyte containing (**a**) 0.0 wt.%, (**b**) 0.5 wt.% (**c**), 1.0 wt.% and (**d**) 2.0 wt.% PMA at elevated temperature.

Lots of previous studies reported that the SEI layer would be rapidly formed at elevated temperature [11,31,33]. Therefore, the surface morphology of the MCMB electrodes after being charged/discharged at 0.1C for 50 cycles at elevated temperature in the base electrolyte containing various PMA concentrations was investigated. Figure 6a presents that the surface of the MCMB electrode tested in the PMA-free electrolyte at elevated temperature was covered with a relatively thicker SEI layer compared with that tested at room temperature (Figure S2a). Additionally, the relatively smooth and even MCMB surface was observed with increasing PMA concentration in the base electrolyte, as depicted in Figure 6b–d. This signifies that the decomposition rate of the PMA additive was also significantly accelerated at elevated temperature.

**Figure 7:**
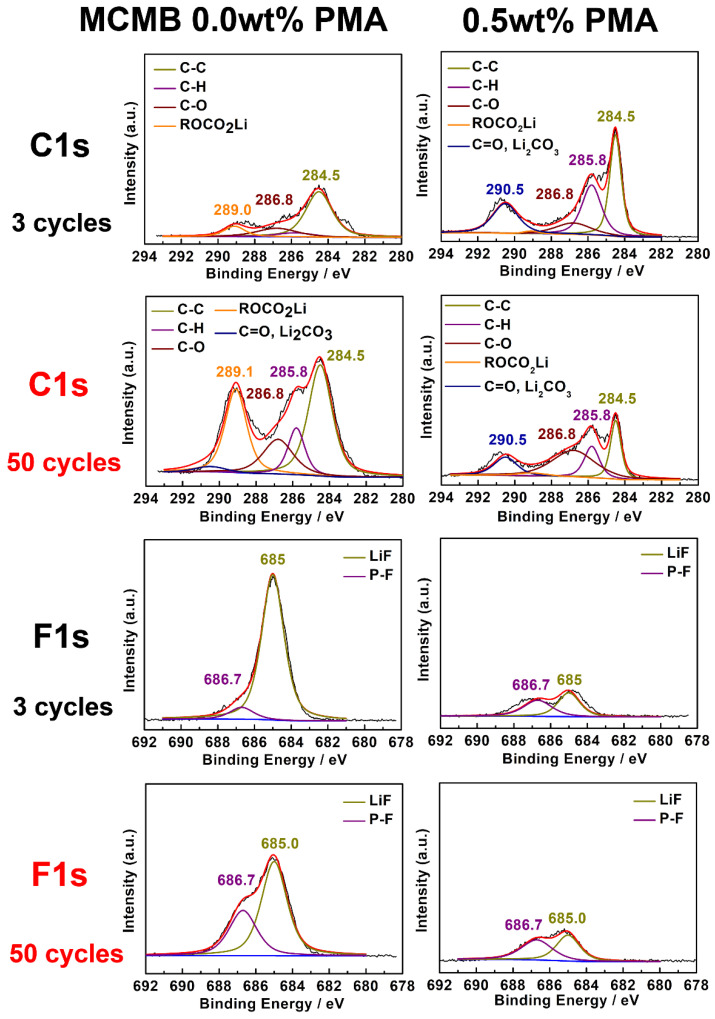
XPS spectra of selected C 1s and F 1s for MCMB electrodes cycled in the base electrolyte with and without 0.5 wt.% PMA additive.

To further investigate the effect of PMA additive on the chemical composition of the SEI films on the MCMB electrode surface, the 3rd and 50th cycled MCMB electrodes at elevated temperature were examined by using XPS analyses, as shown in Figure 7. The selected and detailed XPS results are also summarized in Figure 7 and Figure S3, respectively. The obtained C 1s spectra reveal that the decomposition products of the SEI film consist of C-O and C-C containing groups. As shown in C 1s spectra, 284.5 eV belongs to the sp^2^ carbon-carbon bonding, and it confirms the presence of inherent as graphite nature. The broad peak around 285 to 288 eV can be assigned to the presence of hydrocarbon (C-H) (285.8 eV), which could be possibly derived from alkyl carbonate (R-CH_2_OCO_2_-Li) (286.8 eV) and PVDF (286.0 eV) binder, respectively. Moreover, the peak observed at 286.8 eV is associated with C=O bond of lithium alkyl carbonate when cycled in the base electrolyte with 0.5 wt.% PMA. When compared to the MCMB cycled in the PMA-free electrolyte, the MCMB electrode tested in the base electrolyte containing 0.5 wt.% PMA displays noticeable weaker signals of C-C, R-CH_2_OCO_2_-Li, C=O bonds. Moreover, C=O located at binding energies of 289.0 and 290.5 eV mainly corresponds to Li_2_CO_3_ and ROCO_2_-Li, which are generally regarded as a pair of the essential decomposition products of PMA [34]. Interestingly, it can be found that the intensity of the Li_2_CO_3_ for the MCMB electrode cycled in the base electrolyte in the presence of PMA is relatively weaker when compared to that of the MCMB electrode test in the PMA-free electrolyte. This clearly reveals that the PMA can efficiently retard the formation of organic and inorganic carbonate compounds. 

Figure S3 presents the O 1s spectra of MCMB electrode cycled in the base electrolyte with and without 0.5 wt.% PMA additive. It reveals the main C-O peak at 531.3 eV originating from R-OCO_2_-Li and a relatively weak peak at 532.5 eV due to the formation of Li_2_CO_3_ compound in the SEI film. As for the F 1s spectra depicted in Figure 7, a clear and strong peak appears at 685.0 eV for the MCMB electrode cycled in the PMA-free electrolyte, which would be inorganic LiF resulting from electrolyte salt decomposition. It is found that the intensity of LiF for the MCMB electrode in the PMA-free electrode is relatively higher than that in the 0.5% PMA-containing electrolyte. This signifies that LiF easily grows on the surface of MCMB electrode in the absence of the PMA additive. The large amount of deposited LiF on the MCMB electrode generally leads to high interfacial impedance [17]. In the case of P 2p spectra shown in Figure S3, the peak at 137.2 eV and 133.9 corresponds to that of the LiP_x_OF_y_ and Li_x_PF_y_, respectively. The MCMB electrode cycled in the PMA-free electrolyte shows a much higher amount of lithium alkyl carbonate compounds compared to that in the PMA-containing electrolyte, suggesting that a stable interfacial structure of SEI to prevent the electrolyte from further decomposition could be well established in the presence of PMA additive. This indicates that the addition of 0.5 wt.% PMA can efficiently promote the rapid growth of stable SEI film on MCMB surface and therefore improve its cyclability, especially when operated at elevated temperature.

**Figure 8:**
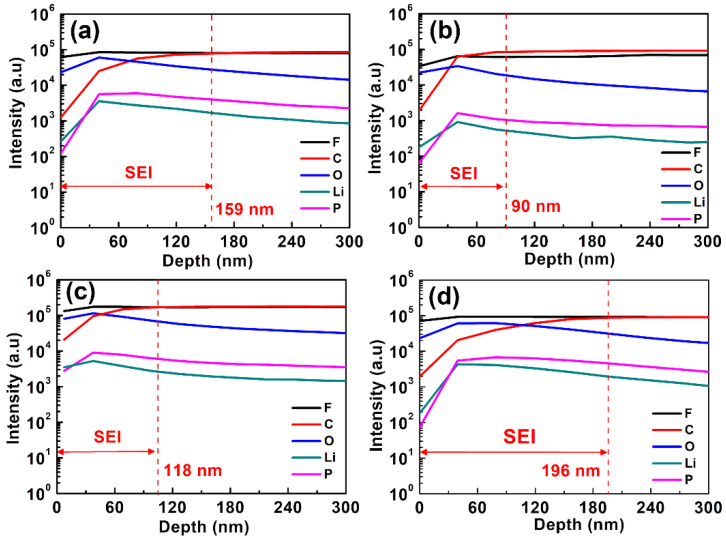
SIMS depth profile of the MCMB electrode with additive-free electrolyte and different concentrations of PMA added electrolyte at 55 °C. (**a**) additive-free electrolyte, (**b**) 0.5 wt.% (**c**) 1.0 wt.% and (**d**) 2.0 wt.% of PMA additive.

To investigate the deep profiles of composition for the SEI layers formed on MCMB electrodes, SIMS analyses were conducted. Figure 8 depicts the content of C, F, O, Li, P elements of the MCMB electrodes charged/discharged in the base electrolyte containing various concentrations of PMA additive for consecutive 50 cycles. According to the variation in depth of the C, F, O, Li, P signals, the SEI thickness on the MCMB surface could be approximately evaluated. The thickness of the SEI layer is estimated to be 110, 36, 45 and 60 nm for the MCMB electrode cycled in the base electrolyte containing 0, 0.5, 1 and 2 wt.% PMA, respectively. It is interesting to note that MCMB electrodes cycled in the base electrolyte containing 0.5 and 1 wt.% of PMA exhibit the SEI layer in the thickness of around 16~18 nm. Hence, it is noteworthy to mention that the addition of 0.5 and 1 wt.% of PMA can help to grow the thin SEI layers on the MCMB surface when compared to that with PMA-free and 2 wt.% PMA-containing electrolytes.

**Figure 9:**
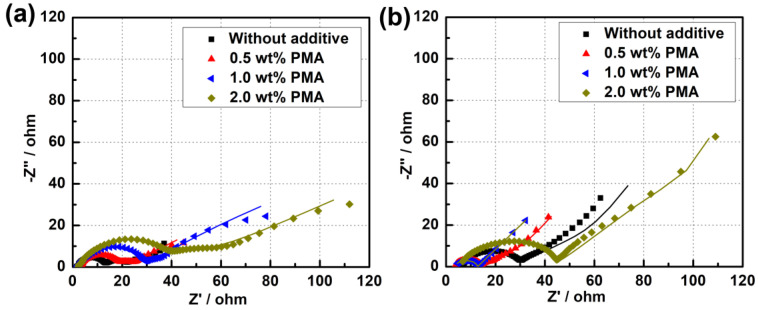
Nyquist plots of MCMB electrodes after cycled in the base electrolyte containing different concentrations of PMA additive for (**a**) 3 cycles and (**b**) 50 cycles at 55 °C.

Additionally, EIS measurements for the MCMB electrodes cycled in the base electrolyte containing different PMA concentrations at elevated temperature were carried out and the resultant Nyquist plots are depicted in Figure 9. The inset of Figure S4a is the equivalent circuit model used to fit the obtained EIS spectra [31,34,35]. The equivalent circuit parameters obtained from the Nyquist plots are given in Table 1. R_s_ represents the electrolyte resistance or solution resistance, the resistance at the first depressed high-frequency semicircle was related to the SEI film formed on the electrode surface (R_sei_), and the resistance at the second depressed mid-frequency difference is related to the charge transfer resistance of both cathode and anode (R_ct_), and the inclined line in the lower frequency represents the Warburg impedance (W). After cycling for 50 cycles, it can be found that the R_sei_ value of the MCMB electrode is remarkably decreased from 10.34 Ω to 4.56 Ωwhen only introducing 0.5 wt.% PMA into the electrolyte. Nevertheless, the R_sei_ value of MCMB electrode is observed to be significantly increased with further increasing the PMA concentration from 0.5 wt.% to 2 wt.%. When introducing a large amount of PMA, the increased R_sei_ value at a high concentration of PMA could be ascribed to the severe decomposition of PMA. This EIS result also confirms that the optimized addition of 0.5 wt.% PMA in the base electrolyte can rapidly stabilize the SEI growth and form a compact and thin SEI layer on the MCMB surface. Therefore, it would efficiently promote the electron transfer between the electrolyte and MCMB electrode and facilitate the rapid Li-ion diffusion process, thus resulting in the low R_ct_ and Z_w_ values.

**Scheme 2:**
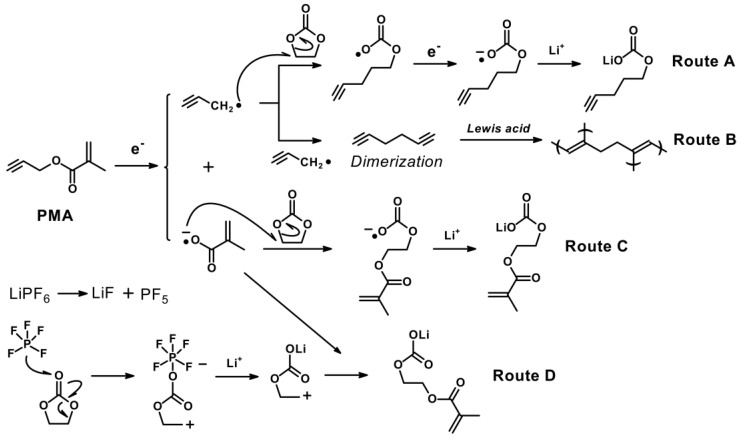
The possible mechanism pathways for the decomposition of PMA on the MCMB electrode.

According to the aforementioned results, we postulated a possible mechanism of SEI formation in the base electrolyte in the presence of PMA, as illustrated in Scheme 2. As depicted in Scheme 2, the dominant SEI components are suggested, which include the Li_2_CO_3_, RCO_2_Li, ROCO_2_Li, etc. Since PMA additives consist of both triple bond and double bond, which are highly prone to decompose and produce both the radical and anion species, and it leads to the uniform formation of the SEI film via route A, B and C, respectively. Simultaneously, the decomposition of the carbonate-based solvent and electrolytes would be effectively suppressed, especially operated at elevated temperature. The triple and double-bonded (CH_2_=CH_2_, CH=CH-CH_2_-COO-CH=CH(CH_3_) compounds and the inorganic (Li_2_CO_3_) SEI products could possibly originate from the PMA additives. As illustrated in Scheme 2, the triple-bond in the PMA compound decomposed by the reduction process would produce the propynyl and methyl methacrylate radicals on the negative electrode. At the initial charge/discharge process, as also shown in Scheme 2, copolymerization reactions of double and triple bonds took place on the negative electrode surface, and it forms a stable SEI film before the electrolyte decomposition. The humidity sensitive LiPF_6_ electrolytes exhibit inferior thermal stability at elevated temperatures, and it can produce the PF_5_, HF and LiOH combined with the trace amount of H_2_O as shown in Equation (6) [36].

LiPF_6_ + H_2_O → PF_5_ + HF + LiOH (6)

Moreover, LiPF_6_, LiBF_4_, and LiAsF_6_ have been shown to generate the Lewis acid PF_5_, BF_3_, and AsF_5_ which are known to catalyse the ring-opening polymerization of poly (ethylene carbonate) (PEC) (as shown in route D) or poly (ethylene oxide) (PEO)-like products with accompanying production of CO_2_ gas [30]. On the other hand, DEC-based electrolyte attacked by PF_5_ is decomposed, resulting in producing HF by side reactions [37].

Due to the copolymerization reactions, the reduction potential of PMA is higher than that of the base electrolyte, therefore the stable SEI film formation by PMA could also protect MCMB electrode from HF attack and result in improved cyclability. On the other hand, part of the propynyl and methyl methacrylate radicals react with EC and Li cations to form lithium alkyl carbonates. Therefore, an excessive amount of PMA added to the electrolyte system causes additional side reactions, which would result in the reduced cycling performance of LIBs [36,38]. The XPS results in Figure 7 also further support and confirm the SEI products formed by the proposed reaction mechanism.

## 4. Conclusions

In summary, the PMA was successfully used as an electrolyte additive to stabilize the SEI formation on MCMB electrode for LIB applications. It is found that the compact and thin SEI layer was formed on MCMB electrode surface when introducing optimized 0.5 wt.% PMA in the base electrolyte. The MCMB electrode cycled with 0.5 wt.% PMA-containing electrolyte exhibited an impressive discharge capacity retention of 86.9% (after 100 cycles, 0.1C rate), and 90.3% (after 50 cycles, 0.1C rate) at room temperature and 55 °C, respectively. The improved cyclability of MCMB electrode in the presence of 0.5 wt. % PMA was ascribed to the formation of a stable and thin SEI layer. The formation of the stable SEI layer was then suggested via a rapid copolymerization reaction of PMA compounds. Therefore, in view of these results, PMA can be regarded as an efficient electrolyte additive for stabilizing the SEI layer on MCMB anodes in LIBs.

## Figures and Tables

**Table 1:** EIS fitting results obtained from Nyquist plots of MCMB.

PMA Content (wt.%)	After 3 Cycles				After 50 Cycles			
	R_s_ (Ω)	R_sei_ (Ω)	R_ct_ (Ω)	Z_w_ (Ω)	R_s_ (Ω)	R_sei_ (Ω)	R_ct_ (Ω)	Z_w_ (Ω)
0.0	2.167	9.837	7.101	49.54	6.68	10.34	10.41	84.19
0.5	3.046	14.04	4.432	96.1	3.72	4.60	4.39	43.40
1.0	2.106	26.43	4.993	107.1	3.45	7.13	2.16	122.00
2.0	2.487	34.65	10.49	305.6	5.25	22.91	15.30	188.90

## Data Availability

Not applicable.

## Supplementary Materials

The following supporting information can be downloaded at: https://www.mdpi.com/article/10.3390/polym14214491/s1, Figure S1. FE-SEM images of (a) Bare MCMB, MCMB material (b) after charge/discharge tests of 100 cycles using additive free electrolyte (c), 0.5 wt.% (d), 1 wt.% (e) and 2 wt.%; Figure S2. FE-SEM images of MCMB anodes after 100 cycles (a) without additive and (b) 0.5 wt.% PMA. Figure S3. XPS spectra of C 1s, F 1s, O 1s and P 2p for MCMB cells containing 0.0 and 0.5 wt.% PMA additive in electrolyte at 3 cycles and 50 cycles; Figure S4. The electrical equivalent circuit (EEC) was employed for simulating the obtained EIS spectra. Table S1. Comparative results of commonly used electrolyte additives to stabilize the graphite anode
