# Supplementary material for: Enhanced Thermal Stability of Mesoporous Carbon Microbeads-Based Lithium-Ion Batteries by Propargyl Methacrylate as Electrolyte Additive
Source: Polymers (Basel). 2022 Oct 24;14(21):4491. doi: 10.3390/polym14214491 (PMC9656890; doi:10.3390/polym14214491)
Supplement: Supplementary file 1 [file polymers-14-04491-s001.zip › polymers-1969913-supplementary.pdf]

Enhanced Thermal Stability of Mesoporous Carbon Microbeads-Based Lithium-Ion Batteries by Propargyl Methacrylate as Electrolyte Additive

Yu-Ruei Kung*[,1], Jing-Tang Su,[1] Chiung-Cheng Huang,[1] Yaoming Xiao,[2] and Jeng-Yu Lin*[,3]

*Corresponding authors

[1] Department of Chemical Engineering and Biotechnology, Tatung University (TTU), 40. Sec.3, Zhong Shan N. Rd., Taipei, 104327, Taiwan (R.O.C.)

[2] College of Chemical Engineering and Materials Science, Quanzhou Normal University, Quanzhu 36200, PR China

[3] Department of Chemical and Materials Engineering, Tunghai University (THU), No. 1727, Sec. 4, Taiwan Boulevard, Xitun District, Taichung City 407224, Taiwan (R.O.C)

E-mail: yrkung@gm.ttu.edu.tw; jylin@thu.edu.tw

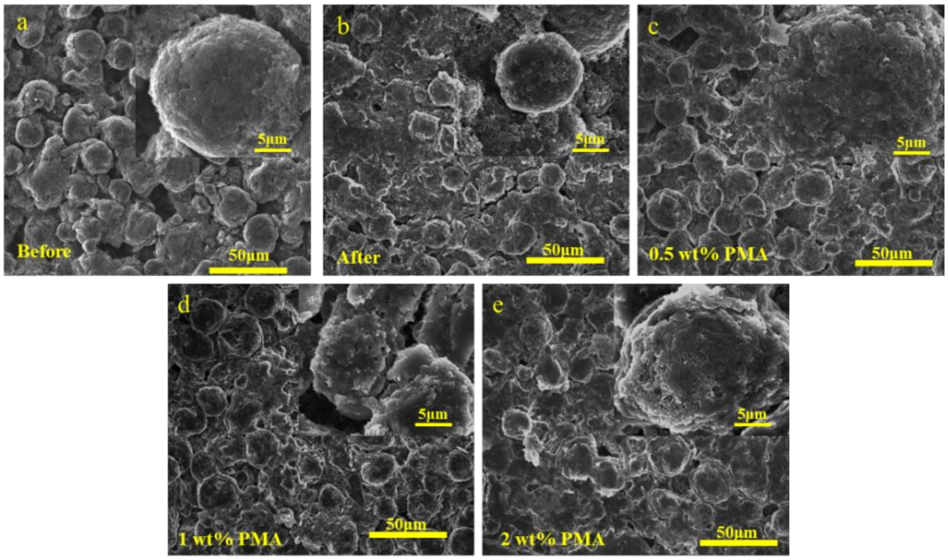

Figure S1. FE-SEM images of (a) Bare MCMB, MCMB material (b) after charge/discharge tests of 100 cycles using additive free electrolyte (c), 0.5 wt.% (d), 1 wt.% (e) and 2 wt.%.

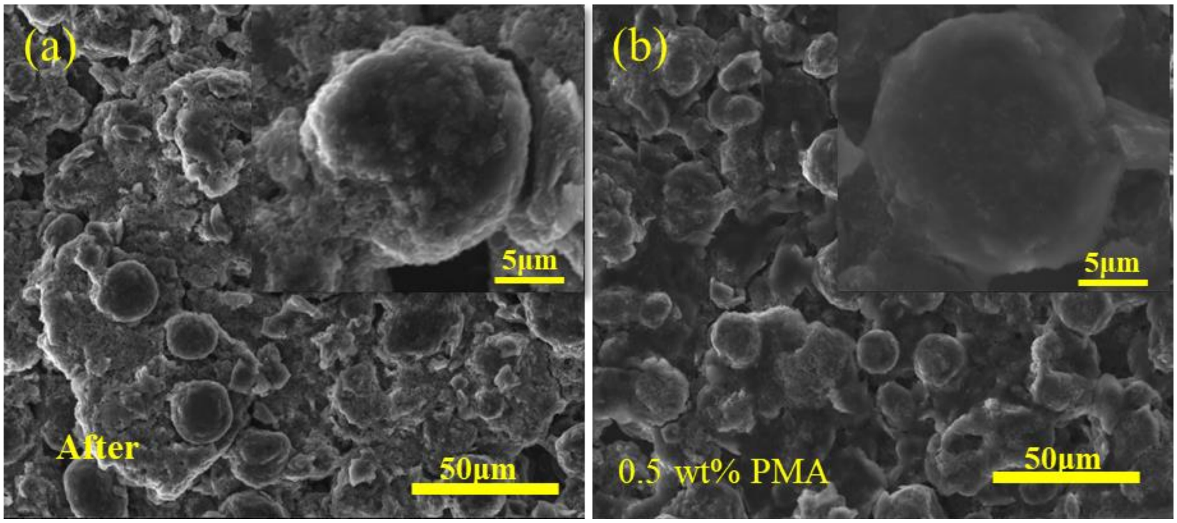

Figure S2. FE-SEM images of MCMB anodes after 100 cycles (a) without additive and (b) 0.5 wt.% PMA.

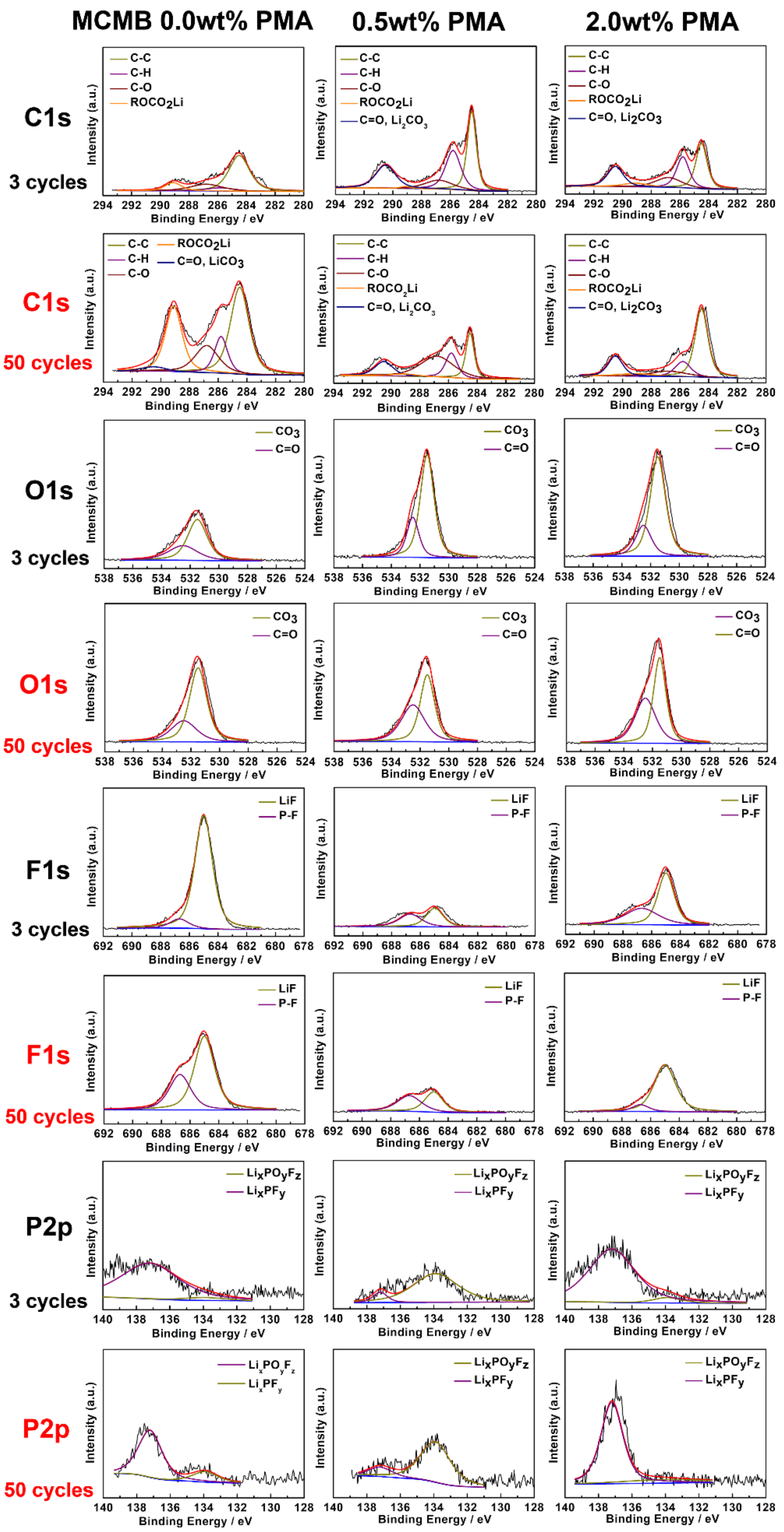

Figure S3. XPS spectra of C 1s, F 1s, O 1s and P 2p for MCMB cells containing 0.0 and 0.5 wt.% PMA additive in electrolyte at 3 cycles and 50 cycles.

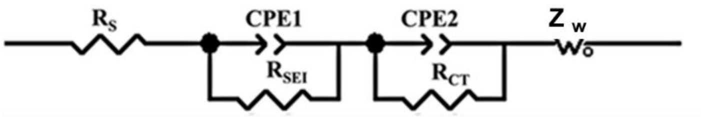

Figure S4. The electrical equivalent circuit (EEC) employed for simulating the obtained EIS spectra.

Table S1 Comparative results of commonly used electrolyte additives to stabilize the graphite anode

Electrolyte composition	Kind System	Additives	Performance	Ref.
1.0 M $LiClO_4$ in FEC/PC/EC (1:3.5:3.5, v/v/v)		Fluoroethylene carbonate (FEC)	Improved cyclability	[10]
1.0 M $LiAsF_6$ in EC-DMC (1:1, v/v)	Carbonate	5wt% Vinylene carbonate (VC)	Higher coulombic efficiency	[11]
1.0 M $LiPF_6$ in PC/DEC (1/1, v/v)		2(5H)-Furanone	Lower interphase resistance	[12]
1.0 M $LiBF_4$ in PC/EC/EMC (1:1:3, v/v/v)	Borate	1-5mol % Lithium bis(oxalato)-borate (LiBOB)	Improved cyclability	[13]
1.0 M $LiPF_6$ in PC/DMC (1:1, v/v)		3,5-bis(trifluoromethyl) phenylboronic acid	Reversible intercalation	[14]
1.0 M $LiClO_4$ in PC		10-20% Vinyl ethylene sulfite (VES)	Reversible intercalation	[21]
1.0 M $LiPF_6$ in EC/PC/EMC (1:1:3, v/v/v)		1wt% Butyl sultone (BS)	Improved cyclability	[22]
1.0 M $LiPF_6$ in PC/ EC (1:1, v/v)	Sulfur-containing system	1,3,2-dioxathiolane-2,2-dioxide (cyclic sulfates)	Reversible Higher coulombic efficiency	[23]
1.0 M $LiPF_6$ in PC-EMC (1:1, w/w)		1,3-Propanesultone (PS)	Reversible performance	[24]

1.0 M $LiPF_6$ in PC	2-6wt% ethylene sulfate (DTD)	Lower interphase resistance/ Improved cyclability	[25]
